# Trust and inclusion during the Covid‐19 pandemic: Perspectives from Black and South Asian people living with dementia and their carers in the UK

**DOI:** 10.1002/gps.5689

**Published:** 2022-02-09

**Authors:** Megan Armstrong, Narin Aker, Pushpa Nair, Kate Walters, Yolanda Barrado‐Martin, Nuriye Kupeli, Elizabeth L. Sampson, Jill Manthorpe, Emily West, Nathan Davies

**Affiliations:** ^1^ Research Department of Primary Care and Population Health, Institute of Epidemiology and Health Care University College London (UCL) London UK; ^2^ Marie Curie Palliative Care Research Department, Division of Psychiatry University College London London UK; ^3^ King's College London London UK

**Keywords:** carers, Covid‐19, dementia, ethnicity, qualitative

## Abstract

**Introduction:**

People from ethnic minority backgrounds living with dementia are more likely to be diagnosed later and have less access to health and social care support than their White counterparts in the United Kingdom (UK). Covid‐19 has exacerbated health inequalities and diminished trust from underserved communities in the government and health services. The wider aim of the study was to explore the impact of covid‐19 on Black and South‐Asian people living with dementia and their carers as well as exploring the experiences of dementia care. The present paper specifically explores their views on trust and mistrust using an ecological model.

**Method:**

Semi‐structured interviews were conducted with 11 family carers and four people living with dementia from South Asian or Black communities living in the community. Thematic analysis was used to analyse data.

**Design:**

An exploratory qualiative design was used.

**Results:**

Four main themes were developed exploring trust at the structural, organisational, community and individual level. At the structural level, participants discussed the inequity of Covid‐19, some lack of trust in the UK Government and confusion in its messaging, and the anxiety sometimes leading to curtailment of media usage. At the organisational level, there was some evidence of a perceived lack of person‐centred and culturally sensitive care from healthcare professionals, as well as concerns around care homes as places of safety. At the neighbourhood community level, participants discussed both a distrust as well as a strengthening of relationships and, at the individual level, factors such as knowledge of services, identity, and faith influenced their experience of the pandemic.

**Conclusions:**

People living with dementia need support at all levels and this study highlights how the pandemic impacted each level. Ways to improve trust in the Government and health professionals alongside culturally adapted health messaging should be explored. Alongside this, an examination of how cultural values and norms may influence help‐seeking responses to dementia and increase trust in services may be helpful post‐pandemic.

## Key points

- The pandemic impacted people from ethnic minority backgrounds living with dementia and their carers at each level of the socioecological model
- A lack of trust in the Government and an increase in anxiety due to the media was reported
- There was a perceived lack of person‐centred and culturally sensitive care from healthcare professionals and concerns around care homes as places of safety
- Participants relationships with their community, knowledge of services, identity, and faith influenced their experience of the pandemic

## 1 INTRODUCTION

Dementia is a syndrome affecting growing numbers of individuals and their carers globally^1^ and the impact of the Covid‐19 pandemic on them is being increasingly scrutinised.^2^ In 2019, more than 920,000 people in the United Kingdom (UK) were living with dementia, and a third of the UK population will likely care for someone living with dementia at some time in their lives.^3^ The growing ethnic diversity of the UK's population highlights the importance of ensuring that the inequalities affecting people living with dementia and carers, in terms of insufficient or poor care and treatment, are not compounded by racial or cultural disadvantages. The experiences of minority ethnic groups during the Covid‐19 pandemic have been explored in terms of risks^4^, ^5^ and health behaviours^6^ in the UK context; this present paper seeks to add to this evidence with analysis of interview data from people living with dementia and their carers in the community during the first year of the pandemic.

Health and social inequalities facing ethnic minority groups are well evidenced^7^ and dementia is no exception. People from ethnic minority groups living with dementia are more likely to be more cognitively impaired, with a longer duration of memory loss, and less likely to be prescribed medication to help manage symptoms.^8^, ^9^ People living with dementia from ethnic minority groups are often diagnosed at a later stage and present to specialised services less often than White British counterparts.^10^, ^11^ The main barriers to accessing care for this population stem from inadequacies in health education provision and other services, which compound limited choices, poor cultural awareness and lack of respect for diversity.^12^ Furthermore, people living with dementia and carers from ethnic minority groups both fear and face stigma by using health and social care systems leading to a lack of trust of such services.^12^

For individuals and their families navigating the dementia trajectory, Covid‐19 posed additional challenges and amplified many problems. Not specific to ethnicity, these challenges often led to a worsening of dementia symptoms, isolation, disrupted routines and reduced access to face‐to‐face dementia services.^13^, ^14^ Although many carers showed resilience during the national lockdown periods of severe social distancing at the heights of the pandemic, many experienced increased anxiety and depression.^15^ Those who had limited support networks seemed to be at greater risk of feeling an increased sense of burden.^16^

The pandemic disproportionally impacted people from ethnic minority groups across the globe, with increased severity and mortality risks.^17^, ^18^ Explanations for the inequalities related to Covid‐19 included higher prevalence of pre‐existing conditions and the impact of social determinants of health (e.g., poorer living environments, less opportunity to work from home).^19^ This is despite evidence (prior to vaccine roll out) that people who were Black British or British South Asian were more likely to engage in Covid‐19 preventative behaviours (e.g., hand‐washing, facemask wearing, and social distancing) than White British people, which was mediated through a higher fear of Covid‐19 and lower political trust.^20^

Diminished trust in governments and health services is emerging as an important legacy of the pandemic in many countries^21^; however, it is likely these trust issues are longstanding amongst these groups and COVID‐19 has brought these issues to light whilst exasperating them. Trust in scientists in the UK remains high, particularly among older people and those who are higher educated,^22^ but by the end of 2020 there was an increase from 42% to 68% in the proportion of the public who believed that the governmental response to Covid‐19 had been confusing and inconsistent.^23^ Research has already highlighted how a lack of trust in UK‐based authorities has led to a low Covid‐19 vaccine update in vulnerable populations.^24^, ^25^, ^26^ To reduce health inequalities, it is integral to explore trust in Governments, health services and communities and how it can be improved.

The impact of the pandemic on those living with dementia in the UK has been previously explored, but few qualitative studies have accessed participants from minority ethnic groups. This present paper reports on data collected as part of (anonymised), aimed at developing an evidence‐based decision tool to support family carers and people living with dementia when making difficult healthcare decisions during the pandemic. As part of this project, we interviewed people from South Asian and Black ethnic groups living with dementia in the UK and found that the pandemic had increased their feelings of fear and anxiety, impacted negatively on their diet and eating habits, compounded feelings of isolation and curtailed their support networks and health care.^27^ It was evident the pandemic was influencing people's confidence in various institutions and required an in‐depth exploration through a socio‐ecological model to capture the interplay between individual, relationship, community, and societal factors influencing trust.^28^

## 2 AIM

The wider aim of the study was to explore the impact of covid‐19 on Black and South‐Asian people living with dementia and their carers living in the community as well as exploring the experiences of dementia care. The present paper specifically explores views on trust and mistrust using an ecological model.

## 3 METHOD

### 3.1 Design

An exploratory qualitative design was used with semi‐structured interviews; reporting was guided by the Standards for Reporting Qualitative Research framework.^29^

### 3.2 Participants

Participants were recruited from a range of sources, including those involved in previous research, GP Practices, online dementia research recruitment websites (such as Join Dementia Research), social media (e.g., Twitter), and two local organisations. We also approached two other organisations (Together in Dementia Everyday (TIDE) and a local Alzheimer's society, but no participants were identified from these. None of the organisations were specifically for ethnic minority communities but were based in areas with high proportion of people from ethnic minority groups.

Recruitment took place primarily from in and around the Greater London metropolis. The study was approved by (anonymised) Research Ethics Committee (17623/002). Interviews took place between July and September 2020.

#### 3.2.1 Inclusion criteria

- People of South Asian or Black ethnicity providing unpaid care to someone living with dementia.
- People over the age of 65 living with dementia of South Asian or Black ethnicity.

#### 3.2.2 Exclusion criteria

- Bereaved/former carers <6 months ago.
- People living with dementia who had been diagnosed <6 months ago.
- People lacking mental capacity to provide informed consent to be interviewed.

### 3.3 Recruitment

Local and national dementia and carer networks initially approached possible participants or they were contacted directly by the research team. Those interested in participating were asked to contact the researchers directly or agreed to be sent a copy of the study advertisement, study information leaflet and consent form (via email or post as preferred). Interested participants were then telephone screened against the study criteria and given an opportunity to ask questions. Interpreters were able to be used if needed.

Consent forms were completed electronically and returned to the research team via email, where possible. These were then countersigned by the researcher and returned to the participant. The consent forms were adapted in plain English for people living with dementia. If participants were unable to complete the consent form electronically, consent was taken verbally and audio‐recorded prior to the start of the interview, with confirmation of this then posted to participants.

Researchers trained in assessing mental capacity consented participants. We followed the principles of the Mental Capacity Act to assess capacity. Participants were screened for mental capacity over the telephone or using video technology; ascertaining whether they understand the information pertaining to the study, can weigh up the risks/benefits of taking part, can retain the information and can communicate their decision and consent to take part.

### 3.4 Data collection

Individual semi‐structured interviews were conducted remotely over telephone (*n* = 13) or via secure internet video (Microsoft Teams, *n* = 2). Interviews were conducted by (Anon; *n* = 14), an academic GP who is British South Asian) and (Anon; *n* = 1, a researcher who is White British). Interviews lasted approximately 1 h (range: 35–75 min) and were audio‐recorded. The interview topic guide was developed with the input of the research team, including our patient and public involvement (PPI) representatives (which included people from ethnic minority backgrounds), and was modified as interviews progressed (See Supporting Information S1). The topic guide focused on the impact of the Covid‐19 pandemic on Black and South Asian carers and people living with dementia, considering daily routines, eating, and drinking, wellbeing, access to dementia services, healthcare and social care, and exploring what extra support they would have found useful. At the end of each interview, we collected demographic data on age, gender, marital status, country of birth, first language, education level and occupation. Ethnicity was self‐defined by participants. We collected demographic information which included ethnic group (using the UK consensus categories), and country of birth. All the participants who reported birth from African countries defined themselves as south Asian. Audio‐recorded interview data were transcribed verbatim, pseudonymised, and checked for accuracy.

### 3.5 Data analysis

Thematic analysis guided by Braun and Clark's framework^30^ was used to identify, analyse, and report themes. Line‐by‐line coding was undertaken in NVIVO 12.^31^ An inductive approach was taken, where different members of the team read several transcripts multiple times to identify codes from the data. Codes were then synthesised into the themes and subthemes and discussed between the whole team to explore and agree meanings and interpretation over several meetings. A socio‐ecological model^28^ was applied to the codes when it became evident that the codes were reflecting individual, relationship, community, and societal factors.

## 4 FINDINGS

### 4.1 Participants

We recruited 15 participants in total. As this is an underserved group it can be challenging to recruit large numbers. At 15 participants, we could not identify further participants to take part despite using multiple sources and snowballing methods. However, towards the later interviews little new information was being revealed in the interviews. Of the four participants living with dementia, two were female and two male, ranging in ages from 66 to 88 years, and all had been born outside the UK. Of the 11 carers, five were caring for a spouse and six for a parent. Ten carers were female and one was male, ranging in ages from 29 to 85. Of the 15 participants, seven were of Black ethnicity (all were Caribbean) with English as their first language and eight were of South Asian ethnicity (seven Indian and one Pakistani) with English as their second language. All participants had some further education with eight having degrees or equivalent (see Table 1). In the quotations below, P denotes person living with dementia and C denotes carer.

**TABLE 1:** Demographic characteristics of participants

		PLWD (*n* = 4)	Carers (*n* = 11)
Age	Under 50	0	1
	50–59	0	4
	60–69	1	1
	70–79	2	3
	80+	1	2
Gender	Female	2	10
	Male	2	1
Ethnic group	Black	2	5
	South Asian	2	6
Country of birth	India	1	3
	Jamaica	2	0
	Barbados	0	1
	Kenya	0	1
	Trinidad and Tobago	0	1
	Uganda	1	1
	UK	0	4
First language	English	2	5
	Indian languages (Bengali, Gujarati, Punjabi, Urdu)	2	6
Age left education	16 and under	0	0
	17–20	0	4
	Over 20	4	7

### 4.2 Themes

**FIGURE 1:**
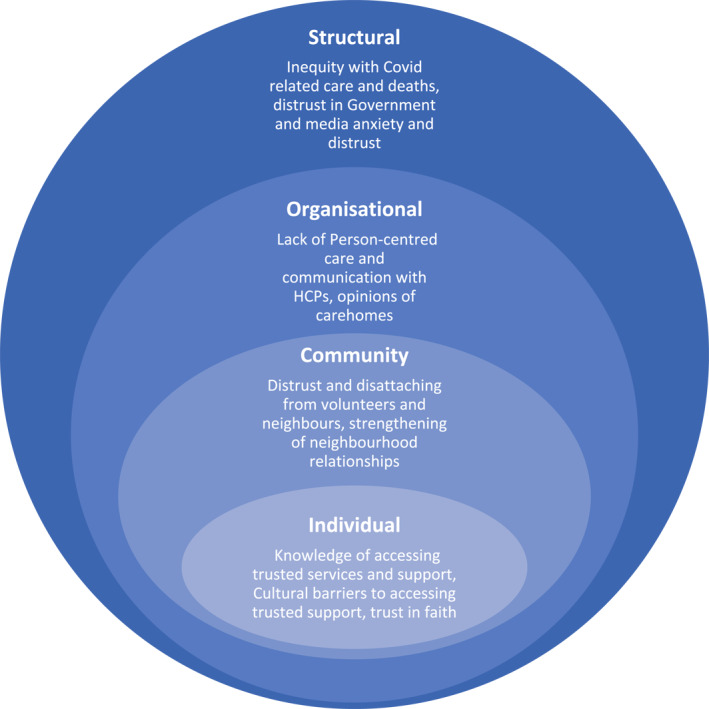
Socioecological diagram of the experiences of people living with dementia and their carers from ethnicity minority groups during Covid‐19

All those interviewed reported feeling some degree of lack of trust and a feeling of exclusion at various levels: individual, the community (neighbourhood and groups), organisational (e.g., NHS) and structural (e.g., Government, social norms). Whilst specific questions were asked regarding Government, media and support, the issue of trust came up organically by the participants throughout the whole of the interviews. These findings are presented using a socio‐ecological model (see Figure 1).^32^

### 4.3 Structural level

#### 4.3.1 Inequality with Covid related care and deaths

Nearly all participants were aware that those from an ethnic minority were more likely to catch the virus and trusted this information. The increased anxiety among some participants:

When that started to come out, yes. That did start to worry me when they said that black and ethnic minority could contract it more than others. That did start to worry me (Carer of a parent, black ethnicity C7).

Some perceived this inequality was driven by the fact that people from ethnic minorities are disproportionally from poorer backgrounds, have less access to services, and are more likely to be in frontline jobs and unable to work from home:

I should be looking into that statistic more, from what I've read it's more people from ethnic backgrounds have mostly… Specifically they're from poorer backgrounds and then that means less access to certain things, maybe healthcare and stuff like that (Carer of a parent, Black ethnicity C7).

Participants handled this knowledge differently, with some preferring to protect the person they were caring for from knowing by not discussing the greater impact Covid‐19 was likely to have to their ethnicity group:

…but I do think that didn't help. Because that's going to create anxieties within families that I don't think there needed to be… They [Person with Dementia] just know it's a bad virus and you're not supposed to go out. And you could catch it and it can make you ill. But they didn't realise that it was going to affect one group more than the other. Because I think that could be quite distressing (Carer of a parent, Black ethnicity C1).

#### 4.3.2 Distrust in government

Many participants expressed some distrust and lack of confidence in the Government's handling of the Covid‐19 pandemic, with many questioning the sufficiency of Government rules and guidance in preventing infection. The lack of trust stemmed from a variety of sources such as not feeling represented in Government, news of Government officials breaking the rules, and a sense of being forgotten about. This lack of trust in the Government seemed more pronounced in the participants who were Black.

One participant described finding the advice confusing:

I've had more clarity, excuse me for being sarcastic, but I've had more clarity from my five‐year‐old grandchild than I did from the government. It was ridiculous. I'm sorry it was ridiculous (Carer of a parent, Black ethnicity C7).

Some reasons for this lack of trust were feeling the Government was making reactive instead of proactive responses, such as bringing in rules and guidance (e.g., lockdown, masks) too late, reducing restrictions too early and not providing sufficient protection from infection (e.g., Personal Protective Equipment) for Covid‐19:

I think because the mask came in later, when it probably should have been a bit earlier. And I think looking back and thinking, they should have done this, they should have done that. It didn't give you a lot of confidence in the government. So, really, I felt that as time was going on, you just had to use your own common sense (Carer of a parent, Black ethnicity C10).

Likely due to not trusting the Government, many participants chose to go above and beyond the guidance provided by engaging in preventative behaviours that were not yet compulsory such as mask wearing in public:

I don't think I've really followed the government advice, as in I've been doing more than what they've been asking. So, with masks now having to be worn on public transports since mid‐June, I've been doing that all the time since the lockdown…just using my own judgment, just being over cautious (Carer of a parent, Black ethnicity C4).

The lack of trust may have stemmed for some from evidence that Government officials had not adhered to the rules themselves. Many, particularly the four people living with dementia interviewed, felt that the Government only cared for people like themselves (e.g., White and affluent) and not for people like them:

Well that's what the government, the people are… What's the word? They get preferential treatment. They know each other. Favourite teams. Family. So it's something I know so I don't rest on it because they only do it for themselves. Advantage (Person living with dementia, Black ethnicity P2).

Some participants felt let down by the Government and felt that the Government should provide more support for people (e.g., financially):

The government should provide the support. Financial. Most thing I know is for the carer [themselves]. If I get relief, somebody comes out and then come back. That type of support (Carer of a spouse, South Asian ethnicity C5).

Another participant living with dementia of Black ethnicity said that they knew Black ethnic minorities would be impacted before it was even announced because this group was not represented enough at higher levels where decisions are made:

You didn't see a Black face. Yes, you see Asian faces. This is when in the government. You didn't see a Black face (Person living with dementia, Black ethnicity P4).

For carers, in addition to feeling unrepresented and not cared for by the Government, in terms of both their carer status and their ethnicity, some did not feel acknowledged during the pandemic either, which likely impacted on how they viewed the Government response:

We weren't given any special treatment like the NHS staff…. They deserve to be prioritised, but I think carers kind of come under that category because we're looking after the ill people as well (Carer of a parent, Black ethnicity C1).

Others were more accepting and thought that people making decisions (e.g., politicians) could not be certain on how to act due to the unpredictable nature of the pandemic:

Well, I think it will be because, as far as I understand, nobody knows the source of these things. I think it's a guessing game going on. They haven't got the proper source of it (Person living with dementia, Black ethnicity P2).

#### 4.3.3 Media anxiety and distrust

The impact of media coverage of the pandemic on people living with dementia was seen as negative by many carers. Many participants found the television news worrying and said that it escalated their anxiety, which led to carers actively trying to reduce watching it and advising or not letting the person living with dementia watch it:

Yes, the news used to make me worried, and I said, you know what, I'll just stop watching it? And I just stopped watching it (Carer of a spouse, Black ethnicity C8).

***Interviewer:*** And do you think the coronavirus situation made them quite worried, your parents?

***Participant:*** Yes. That's why I had to say to Dad, stop watching the news every minute it's on. I had to limit it (Carer of a parent, Black ethnicity C10).

No, definitely anxiety. Definitely, it did. Because also he couldn't express, so if he wants something on the news, and actually I was trying to limit the news, but because he had stopped watching everything else, he would just watch the BBC news, and it was repetition. And that's when I would try to take him for a drive or sit in the car, just try to break up his day (Carer of a spouse, South Asian ethnicity C2).

There was also some distrust in the media (e.g., news outlets), with some participants referring to the media as *lying* or *sensationalizing*:

I think the media needs to get themselves sorted as well, because they like to sensationalise, but I'm logical enough to see that's stupid (Carer of parent, South Asian ethnicity C6).

A few felt those in the media were not doing their job to provide the public with accurate or clear information:

What about those people who didn't have anybody during that period, but was hearing that news all the time over and over again. What would that have done to them? So I think for me, that's it. Just the way in which news is relayed and the guidance needing to be a lot clearer and tighter for us to understand (Carer of a parent, Black ethnicity C7).

Some distrust of the media was expressed in relation to its portrayal of healthcare professionals (HCPs). One person suggested the media portrayed HCPs in a negative light, which did not reflect their experiences:

The nurses, the doctors, the cleaners, they were just wonderful people, and I had no complaint at all. I was well, well looked after. Because, really, after seeing it for yourself, you hear it in the papers, read it in the paper, hear people saying this and that, but it's not when you go through it. They work so hard. They're always on their feet (Person living with dementia, Black ethnicity P2).

### 4.4 Organisational

#### 4.4.1 Lack of person‐centred care and communication with HCPs

Three carers discussed feeling powerless and anxious when the responsibility of caring for their relative changed when they were admitted to hospital. This was intensified due to the prohibition of hospital visiting at the height of the pandemic and the intensity of hospital activity at this time. The lack of communication led to carers feeling disenfranchised and angry with HCPs:

The hospital, to be honest, were terrible. I will be honest. We were outside clapping for carers, I think they were a disgrace in terms of dementia. Absolute disgrace…The actual medical support she got was very thorough. I couldn't fault that. But the problem was they were telling her things. She didn't have a clue what was happening. So, no one communicated to me what was going on (Carer of parent, South Asian ethnicity C6).

The most critical feedback came from some of the South Asian participants of whom many, unlike the Black community, had English as their second language. One participant outlined how they felt the HCPs may have been prejudiced by assuming they could not speak good English due to their ethnicity. Overall, this led to the carers feeling frustrated with the HCPs and less likely to communicate with them:

And then he says, does he speak English? At that point I said to him, should I tell you something? I'll tell you something. He has been in this country since (19)59. He was a bank manager all his life. I was a head teacher. And now I leave it to you to decide whether we speak English or not (Carer of a spouse, South Asian ethnicity C2).

This feeling of prejudice from HCPs led to carers not trusting the decisions the HCPs were making and feeling they had to ‘fight their corner’:

I had to make two important decisions, because two and a half weeks into his stay in hospital, the occupational therapist rings me up and says, ‘we are coming to assess the home tomorrow, because we are discharging him’. I said, ‘what? I said, last night he fainted while I was there, and it was an emergency. There were six nurses there. And you're going to send him home?’ ‘Yes, because he's not cooperating with us. He's not understanding what we are trying to tell him’. I was really horrible to them (Carer of a spouse, South Asian ethnicity C2).

The above quote also appears to show a lack of knowledge of dementia with the HCP saying ‘he's not cooperating with us' further indicating a lack of person‐centred care. When participants did feel understood by HCPs, they perceived the care better, which led to trusting the HCPs and their advice:

The two nurses who understood did magic with him. He would listen to them. He would eat. He would drink with them. He would let them change him. But those who didn't understand… (Carer of a spouse, South Asian ethnicity C2).

Furthermore, explaining options to the carer and encouraging the carer to be part of the decision making was clearly valued by participants and this could be done remotely:

So, this really nice doctor rang me and talked me through the fact that they were going to put her on blood thinner, warfarin. They went through different versions of it with me, and he was actually very good. He helped me make the choice of which blood thinner she needed to be on (Carer of parent, South Asian ethnicity C6).

#### 4.4.2 Opinions of care homes

Beyond hospitals, participants wanted to avoid care homes due a lack of trust in their safety. Some carers feared the risks of acquiring Covid‐19 in such locations as they felt that hospitals were discharging Covid‐19 infected patients there.

The belief is that the people, the elderly people in hospitals, were sent to the care homes, and they had Covid, so they spread it (Carer of a parent, Black ethnicity C1).

Other carers did not trust care homes to provide quality care generally and perceived this would be even worse during the pandemic:

Just the way that they're treated. The cleanliness of some of the places. The cleanliness of the patients, all the residents that are there. Just the general care and love. Just the love and care that's not on the level. And we can understand because you've got 60 odd or 30 odd people to care about (Carer of a parent, black ethnicity C7).

Overall, feeling care homes were being used for Covid‐19 patients and that they were not providing high standards of care could lead to carers feeling care homes were not an option and may isolate or stop carers from trying to access support.

### 4.5 Community

The Covid‐19 pandemic limited dementia specific community services and general community resources. This appeared to amplify a sense of distrust among some study participants who described withdrawing further from the community and not accessing potential support; however, conversely, others described a strengthening of relationships within their neighbourhoods.

#### 4.5.1 Distrust and detaching from volunteers and neighbours

Some participants did not accept support supplied by neighbourhood volunteers as they were concerned about possible criminal intent and did not trust them:

There were a lot of people on the road that were willing to do volunteering and whatever. We didn't take any of the offers up, because I didn't know these people. They were not DBS (Disclosure and Barring Service) checked. Who are the people? They were people on the road that dropped notes to through the letterbox… But because they were all volunteers, I didn't trust anyone. So, I think although it was quite nice that people were willing on their own to do things, I just didn't trust anyone enough (Carer of parent, South Asian ethnicity C6).

Furthermore, the same participant had concerns about the hygiene of the volunteers:

So, I thought, hang on, I don't know these people. Although they're saying that they can make food and bring it around, I don't know what their hygiene is like. I just don't know (Carer of parent, South Asian ethnicity C6).

Some people living with dementia felt others in their neighbourhood were not adhering to the rules, which caused them anxiety:

But when we go out, supposed to keep a distance, but some people don't understand at all, just careless, they're walking. So, naturally, I get upset, they're careless. Or there's some men, you know, probably got it, coronavirus, people… (Person living with dementia, Black ethnicity P2).

Furthermore, one participant described feeling let down by their faith community, due to a lack of communication from them when the pandemic began and from their lack of tangible support for their community:

I think it's just a bit annoying that they've just locked the gates and closed the doors without any communication, and that's been quite hard for the elderly, generally. But what I'm saying is that the temple could have done something once a week, because they had all these elderly people that always used to go to the temple once a week or twice a week. They've obviously closed the doors, and some of the elderly people that I know have been quite isolated. So, I did send to the temple, why don't you do food parcels? Just a bit of roti, a bit of dhal, just drop it around. Just check in on people. But they didn't really take that offer up, which I thought was a bit sad (Carer of parent, South Asian ethnicity C6).

Another participant felt that the contradictory opinions of others about what they should do added more stress and anxiety and led to them cutting off those acquaintances:

Because what was happening is so many friends, people, community, and everybody had a different story to tell me, and I just couldn't take it. For example, “you're not allowed in hospital. God, how will he manage without you?” Then, when I was allowed, I was told, “he is ill, just leave him alone” (Carer of a spouse, South Asian ethnicity C2).

#### 4.5.2 Strengthening of neighbourhood relationships

Others, however, found the pandemic strengthened their relationship with their neighbours by giving more time to get acquainted and build up trust:

The people that are there permanently, you know them, but I've actually gotten to meet neighbours that I didn't… You're too busy. You don't seem them. Neighbours, ‘are you al right?’. Everybody is looking out for each other. That's really nice. And even mum's neighbours, they're really nice. They're always, is your mum all right? Anything, just let me know (Carer of a parent, Black ethnicity C1).

Relationships with neighbours sometimes appeared to have been strengthened through people looking out for each other and mutual aid:

And the neighbours have been very helpful, in the sense of just texting and asking for updates, encouraging for making decisions. My neighbour, [Mr and Mrs X}, they're in their 80s, and they are on all sorts of gadgets for walking and things. But they say that if I ever need help, they'll help me (Carer of a spouse, South Asian ethnicity C2).

### 4.6 Individual

Trust was explored differently at the individual level by exploring how likely individual factors, such as knowledge and culture, were likely to influence accessing services and support that they trusted. Additionally, as explored in detail elsewhere [Authors], we briefly explore here how trust in faith may have supported some of this population.

#### 4.6.1 Knowledge of accessing trusted services and support

How much support people received appeared to largely depend on whether they knew how to access various services and whether they found them acceptable. Below, a participant describes trying to get hold of a letter to allow her to shop at quieter times and before general opening at the start of the pandemic, and how offers of support kept needing to be repeated:

It took time. It never occurred to me to ask them [the council]. They actually emailed it to me. That's another thing. I didn't quite know how to get help. Things kept coming my way, rather than me seeking it (Carer of a parent, Black ethnicity C1).

Some participants said they were not offered anything, although some had, like C1 above and C4 below, received information leaflets mainly from local support organisations:

***Interviewer:*** But no one has offered anything to you yet?

***Participant:*** No, nothing. Nothing (Carer of a spouse, South Asian ethnicity C5).

Those participants who were able to access support or various services found it useful even if it was delivered remotely. Below, a participant talks about appreciating a psychoeducational online programme

So, they have this [name of programme] for carers that help them manage looking after someone with dementia. So, that was extremely helpful. That was eight sessions across eight weeks (Carer of a parent, Black ethnicity C4).

Another participant discussed a system that allowed them to contact their relative living with dementia online and was particularly helpful in linking up with family members in another continent. The participant had come across this system by chance in conversation with another carer and seemed to have trusted this recommendation:

We've recently just put in the Komp, I don't know if you've heard of it. But it's called No Isolation system. It's really brilliant, so we can FaceTime her just to see if she's okay. It's brilliant because my brother in [name of another country] can talk to her et cetera…Plus we can also text her. So we can flash up messages on the screen for her and we can also face‐to‐face the messages….It's a way of us keeping track on her. So last night my check in call with her was on the FaceTime. I was able to say, you all right mum, and she's yes, yes. And it's a good, really good (Carer of a parent, Black ethnicity C7).

One participant described having paid for private healthcare during the pandemic due to the perceived failing of the NHS service. She did not understand what had happened to her parent in hospital as no one had explained her parent's medical treatment in detail to her:

I've paid £250 to see the cardiologist, but I think it was worth it's money. And then, I've paid £300 for that blood test at X Hospital. And I think to pay £500 for someone to tell me, this is what happened… He actually sent me a really nice email and a letter explaining it all, which I shared with my family. And I think that was important (Carer of parent, South Asian ethnicity C6).

#### 4.6.2 Cultural barriers to accessing trusted support

Some participants felt that their culture and how they identify with it meant they were less likely to accept outside support as they believed they should care for their relative living with dementia themselves and this attitude did not appear to have changed over the pandemic:

That's cultural. Because within our culture, because obviously you're non‐white as well, I think we're more for ‘caring for our elders’ ourselves. We don't leave it to other people to do. So for us to just hand over our elders into the care of other people, and then not even check that that care is adequate is just not, it's just not within us. And it's just not an option for us, it really is just not an option for us at all (Carer of a parent, Black ethnicity C7).

However, another participant described feeling that services just assumed they would be able to take on the responsibility of looking after the person living with dementia because of their ethnicity:

***Interviewee:*** I think social services weren't that great. I think, I think it's probably what we have in the Asian community as a social services and such, you know, fundamental organisations, feel that it's up to the family to look after people with any kind of ailment.

***Interviewer:*** And do you feel that was because of your ethnic background?

***Interviewee:*** Oh yes definitely (Carer of a parent, South Asian C11)

Another participant described refusing help and support as a matter of pride even though a trusted friend had made a recommendation:

A friend of mine, she said to me, you're not working, and your parents aren't getting anything financially, so there's an organisation in [name of town], they were offering food boxes and things like that. So, she said to me, would you like it? And to tell you the honest truth, I was hesitant. I said, no, just let someone that needs it more than me. […] And I think it was a pride thing, if I'm honest. I've never had to get anything. I've never had to have a handout, for want of a better word (Carer of a parent, Black ethnicity C10).

#### 4.6.3 Trust in faith

Despite their individual challenges, many participants found comfort in their faith and placed their trust in prayer to help them though this difficult time:

We pray for the family because remember I have the church. So, we on Wednesday we pray for everybody. So in a way although we do it normally before, we pray for people. We pray. In fact whether they're black or white, you know what I mean? So we pray because we're all human beings. So we really prayed (Person living with dementia, Black ethnicity P1).

I think my faith has helped me. Not I think, I know my faith has helped me (Carer of a parent, Black ethnicity C10).

Nonetheless, due to the pandemic, some were unable to engage in their faith practices as much as they would have liked:

I started attending some Islamic lectures on Zoom, and that's it…I didn't fast. I could not fast. No way could I fast, because of looking after him. And then, on Eid day, all this started, his fall and everything (Carer of a spouse, South Asian ethnicity C2).

## 5 DISCUSSION

This study explored the perceived impact of Covid‐19 at various socio‐ecological levels for people living with dementia and their carers of South Asian or Black ethnicity in the UK. People living with dementia need support at all these levels and this study highlights how to some degree the pandemic impacted each level. Nearly all participants expressed limited trust and confidence in the UK Government. Only a couple of participants felt the Government was trying their best, although admitted this was likely not good enough. This lack of trust in the Government seemed more pronounced in the participants who were Black, which is in line with previous research that has found somewhat lower political trust in this minority ethnic group due to a belief that politicians make decisions based on advantaging people who are White.^33^ Despite the limits of their trust in the Government, preventative Covid‐19 related behaviours were adopted and often maintained strenuously in our sample, as found in other studies.^17^ Our findings are therefore somewhat different from those of Hanson et al (2021) who reported their middle class South Asian participants' views of the success of the government's messaging and their high level of trust in the UK government.

A minority of people living with dementia and their carers expressed some lack of trust in the media but more reported finding it anxiety inducing. Some have suggested that the media has consistently portrayed people from minority ethnic groups in a negative or stereotypical way.^34^, ^35^ Accessing accurate and informative information regarding the pandemic and how it affects dementia outside of the media may be a role for HCPs who are well trusted by most minority ethnic people in the UK according to a YouGov survey.^36^ One Taiwanese study found people who received information from HCPs had higher levels of trust in this information and reported improved overall wellbeing than those who received the same information from the media.^37^ Furthermore, research has shown people from ethnic minority groups tend to respond poorly to generic health messages through the media or government, highlighting the need to develop culturally adapted health information.^38^

At an organisational level, Covid‐19 had a big impact on health services, and it may be that with the additional demands of the pandemic, person‐centred and culturally sensitive care was adversely affected, which led to some people living with dementia and their carers feeling marginalised. With one participant in this study, this seemed to be exacerbated when the person living with dementia refused care. A recent study exploring declining care, highlighted the potential of improving hospital treatment for patients living with dementia if HCPs use higher entitlement requests (e.g., telling the patient a task needs to happen by highlighting its importance), followed by requesting permission, and the lowering of contingencies (e.g., making the task sound smaller).^39^ Ultimately HCPs may need to balance encouraging people living with dementia to engage in self‐care or accept care whilst maintaining the right to refuse. Some decision aids, particularly around dementia and Covid‐19, may help people make decisions about their care,^40^, ^41^ including when care services may be a positive choice, and HCPs can advocate the use of these. Such approaches need to be tested for cultural sensitivity.

COVID‐19 impacted local communities globally, causing many to pull together and support those more vulnerable, as is often seen in times of crisis.^42^ Social and religious support can improve wellbeing during challenging and uncertain times,^43^ although these community behaviours tend to decline as emotional and physical resources become depleted.^44^ Some individual factors also seemed to play a role in influencing participants' level of trust and the care received, such as their knowledge of how to access services and support; amplifying the trend for ethnic minority groups being less likely to access dementia services.^8^ ^,^ ^12^ Another individual factor that emerged is how the carer identifies with their culture. Some participants felt it was their responsibility and part of their culture to care for their family or elders, although values and beliefs about the role of the family in caring for the person living with dementia will vary between and within different cultures. The impact this has on carer wellbeing and trust is unclear^45^ and warrants further exploration.

## 6 STRENGTHS AND LIMITATIONS

Ethnic minority groups are often underserved in research, so although numbers are small, this qualitative study is one of the few to have explored how COVID‐19 affected a sample of ethnic minority participants living with dementia and carers in the UK. All but one interview were conducted by a researcher of South Asian ethnicity, which may have helped with ‘insider’ knowledge and trust among participants from that group.^46^ Discussions with the wider study team have enabled a range of perspectives to be included. Applying the Socio‐Economic model allowed the study team to consider how trust was impacted at various levels thereby breaking down future suggestions from individual to societal level. However, limitations of our study are that most of our sample were highly educated, able to use the internet, connected to support networks and could speak English. We acknowledge that there are likely to be additional pressures for those experiencing socio‐economic deprivation and those who do not speak English Our sample may have a selection bias with those agreeing to take part having a general interest in research. Additionally, we could not explore cultural differences across sub‐groups, but only broadly across people who are Black or South Asian.

## 7 FUTURE RESEARCH

Any inquiry into the UK response to Covid‐19 may wish its findings and recommendations to improve trust in the Government and the media. Some have called for a period of reconciliation where it would be acknowledged that ethnic minorities were let down followed by measurable changes.^47^ Alongside this, an examination of how cultural values and norms may influence help‐seeking responses to dementia and increase trust in services may be helpful post‐pandemic.

## 8 CONCLUSION

To our knowledge this is the first study to explore the perceived impact of Covid‐19 on the various socio‐ecological levels for people with living with dementia and their carers who are of South Asian or Black ethnicity in the UK. People living with dementia need support at all these levels and this study highlights how to some degree the pandemic impacted each level. For some, a lack of trust in Government guidelines and anxiety surrounding media reporting, as well as an absence of person‐centred and culturally sensitive care, led to a sense of disenfranchisement. Our data also revealed the presence of highly protective behaviours against Covid‐19 that went beyond current guidelines, potentially causing additional stress and anxiety but enabling a sense of agency and control. Trust needs to be rebuilt at every level for this population and going forward with the learnings around health communication and person‐centred care at times of crisis.

## CONFLICT OF INTEREST

There are no known conflicts of interest.

## ETHICS STATEMENT

The study was approved by UCL Research Ethics Committee (17623/002). All participants gave informed consent either electronically or verbally.

## Supporting information

Supporting Information S1

## Data Availability

Data are available on reasonable request from the authors.
