# Supplementary material for: Trust and inclusion during the Covid‐19 pandemic: Perspectives from Black and South Asian people living with dementia and their carers in the UK
Source: Int J Geriatr Psychiatry. 2022 Feb 9;37(3):10.1002/gps.5689. doi: 10.1002/gps.5689 (PMC9015357; doi:10.1002/gps.5689)
Supplement: Supplementary file 1 — Supporting Information S1 [file GPS-37--s001.docx]

**An Exploration of the impact of Covid-19 on dementia care in Black and Minority Ethnic groups (People with dementia)**

1. How are you//how has your week been? Just to start off – can you tell me what your typical routine/day looks like?
2. **How does the situation with regard to Coronavirus impact your daily life? (e.g. daily routines)**

- How has it affected you daily routine? (ask if in a care home, how it has affected daily life….)
- How has it affected your social/family life? (e.g. birthdays, festivals)
- Have you had any particularly challenging moments?
- How has it affected your wellbeing?
- Have your needs changed?
- Do you think Coronavirus has a greater impact on you, as someone experiencing memory problems from an ethnic minority background?
- What extra support, if any, have you been offered?
- What has helped you maintain your wellbeing during this time?
- Do you know anyone who has been affected by coronavirus (bereavements, able to attend funerals, visiting)
- What do you think about the way in which the coronavirus pandemic has been reported by the government/news? How has it affected you?

1. **Could you describe if and how Corona virus impacts your current situation regarding access to/use of dementia care services and support?**

- How has it affected your access to the GP?
- How has it affected your access to community dementia care services, e.g. dementia hubs/organisations, memory clinic?
- Has it affected your formal caring (if any) arrangements?
- How have you been able to communicate with healthcare teams? How do you feel about this?
- Have you had any discussions about your care during this time and planning for the future (e.g. if you become unwell/advance care planning)?
- What extra support, if any, have you had/been offered? Has this support been appropriate for your specific/cultural needs? (e.g. voluntary services, community support, formal support)

1. **What kind of support would you have liked to have had that are not available due to Corona virus?** (e.g. help from friends/family, help from professionals, help with family tasks like shopping, emotional/psychological support)
2. **Specific questions about eating and drinking in dementia:**

- Since your diagnosis, have you noticed any changes in your eating/drinking? (e.g. flavours, portions, textures; mealtimes)
- What do you think the role of food has in your culture/life? Does food/drink have any religious significance for you? Festivals?
- Is the social/communal aspect of food important to you? (e.g. family mealtimes)

1. **How does the situation with regard to Corona virus impact on your eating and drinking needs?**

- How does it affect shopping for food? How has this affected you?
- How does it affect food deliveries? Food parcels?
- How does it affect availability of food? E.g. cultural foods
- Have you received any help/support with this? (e.g. voluntary, community or formal support)
- Has it caused you to change your diet? If so, how? Has this had an impact on your health?
- Has it affected your mealtimes? E.g. social interactions with others around mealtimes/ family meals? How has social distancing impacted on this?
- Have any cultural events/festivals been affected? If so, in what way?
- What support are you currently receiving with regards to helping you manage food and drink? Is there any other support you would like? In what format/delivery?

***Close***

**Topic Guide - An Exploration of the impact of Covid-19 on dementia care in Black and Minority Ethnic groups (Carers)**

1. **Can you tell me who it is you care for and where they are currently living? What is your daily routine like?**
2. **How has the situation with regards to Coronavirus impacted on how you are able to provide care or support your relative/friend with dementia?** (e.g. daily routines, care home context)
   1. Have you had any particular challenges or challenging moments?
   2. Have you explained to (name of person with dementia) what’s going on? What information have they been given?
   3. Respite services and home care workers – are they are wearing PPE? How has this affected the PwD (e.g. are they reacting distressed etc)
   4. Have you had to make any decisions about their care? If so, why and how?
   5. Do you feel more or less worried about the coronavirus situation in view of your ethnic background? (reports of higher incidence..)
   6. *If relative in a care home*: have you been able to visit your family member with dementia? Have you received sufficient communication from the care home?
   7. Explore socio-cultural impact, e.g. birthdays festivals
   8. Do you know anyone who has been affected by corona virus? (bereavements, able to attend funerals, visiting)
3. **Thinking about the person you provide care and support for, how has (name of person with dementia) reacted to the corona virus situation?**
   1. Has it had an effect on them do you think? (explore impact of news/information etc)
   2. How has it affected their wellbeing? What, if anything, has helped maintain their wellbeing during this situation?
   3. Have their needs changed?
   4. Probe about routine and how this has been affected
   5. What have you found is helpful to them?
   6. Explore role of technologies for keeping in touch, e.g. telephone, video
4. **Could you describe if and how Corona virus impacts your current situation regarding access to/use of dementia care services and support?**

- How has it affected your access to the GP?
- How has it affected your access to community dementia care services, e.g. dementia hubs/organisations, memory clinic, carer organisations?
- How have you been able to communicate with healthcare teams? How do you feel about this?
- Have you had any conversations around advance care planning in view of Covid (i.e. if PwD becomes unwell)?

1. **Now thinking about your needs as a carer. Could you describe if/how the Corona virus has impacted on your current needs and situation?**

- How has it affected your caring arrangements? (e.g. family or formal carers/social care)
- How has it affected your social life / family life?
- How has it affected your wellbeing?
- Do you think the effect for you is heightened, as a carer of someone with dementia from a BAME background?
- What do you think about the way in which the coronavirus pandemic has been reported by the government/news? How has it affected you?
- What do you think about the government’s advice about the pandemic?
- What extra support, if any, have you had/been offered? Has this support been appropriate for your specific/cultural needs? (e.g. voluntary services, community support, formal support)
- What has helped you to maintain your wellbeing as a carer during the Corona virus situation?

1. **What kind of support would you have liked to have had that are not available due to Corona virus? (**e.g. help from friends/family, help from professionals, help with family tasks like shopping, emotional/psychological support)
2. **How does the situation with regard to Corona virus impact on how you are able to support (name of person with dementia) to continue eating and drinking?**

- Can you tell me a bit about your experience of managing meals and food for XX?
- Have you noticed any changes in their eating and drinking recently? Has the Coronavirus situation impacted on this (e.g. stress, anxiety, changes to routine)
- If they are in a care home – how has their management of food and drink been affected? Are they usually involved in their diet? If so, how?
- Do you think food has different roles in different cultures? (explore religious significance, festivals)
- Is the social/communal aspect of food important to you and for the PwD? (e.g. family mealtimes)
- How has COVID-19 affected shopping for food?
- How does it affect food deliveries?
- How does it affect availability of certain foods? E.g. cultural foods
- How have you managed this? Have you received any help/support?
- Has it caused you to change their diet or how you prepare their food? If so, how? What impact has this had? And how do you manage this?
- Has it affected your mealtimes? E.g. family meals? How has social distancing impacted on this?
- Have any cultural events/festivals been affected (e.g. Ramadan)? If so, in what way?
- What support are you currently receiving with regards to helping you manage food and drink? Is there any other support you would like? In what format/delivery?
